# Does Bisphosphonate Increase the Sclerosis of Tibial Subchondral Bone in the Progression of Knee Osteoarthritis—A Propensity Score Matching Cohort Study Based on Osteoarthritis Initiative

**DOI:** 10.3389/fmed.2021.781219

**Published:** 2021-11-22

**Authors:** Mingyang Li, Yong Nie, Yi Zeng, Yuangang Wu, Yuan Liu, Limin Wu, Jiawen Xu, Bin Shen

**Affiliations:** Department of Orthopedics, Orthopedic Research Institute, West China Hospital, Sichuan University, Chengdu, China

**Keywords:** bisphosphonate, subchondral bone morphometry, BVF, Tb.N, Tb.Sp, Tb.Th

## Abstract

Bisphosphonate has great potential in KOA therapy, but whether the anti-resorption mechanism of bisphosphonate aggravates sclerosis of subchondral bone remains unclear. We found that bisphosphonate use did not increase sclerosis of subchondral bone in established KOA, perhaps resolving some concerns about bisphosphonate in patients with KOA.

**Introduction:** Most studies have focused on the protective effect of bisphosphonate on early knee osteoarthritis (KOA) through its anti-resorption mechanism in osteoclasts. However, late KOA has a decreased rate of resorption, which is the opposite of early KOA. The risk of subchondral bone sclerosis in late KOA after using bisphosphonate has not been investigated using morphometry.

**Methods:** Forty-five patients who had ever used bisphosphonate (or 33 patients with current use) were matched with controls through propensity matching methods, including age, body mass index (BMI), sex, health status (12-Item Short Form Survey physical health score), physical activity level (Physical Activity Scale for the Elderly score), vitamin D use, and calcium use. At the baseline and 12-month (or 18-month) follow-up, bone mineral density (BMD) of the tibia and hip was measured by dual-energy X-ray absorptiometry (DXA), and medial tibial subchondral bone morphometry: bone volume fraction (BV/TV), trabecular thickness (Tb.Th), trabecular number (Tb.N), and trabecular separation (Tb.Sp) were calculated based on 3-T trabecular MRI. Data were obtained from the Bone Ancillary Study in the Osteoarthritis Initiative (OAI) project.

**Results:** The yearly percentage change in hip BMD of the current bisphosphonate-use group was significantly greater than that of the non-bisphosphonate-use group (0.7% vs. −1%, *P* = 0.02). The other outcomes (BV/TV, Tb.N, Tb.Sp, Tb.Th, tibia medial BMD, and tibia lateral BMD) between the two groups presented no significant difference. The non-bisphosphonate-use group experienced a significant increase in Tb.Th [2%, 95% CI = (1%, 4%), *P* = 0.01], while the bisphosphonate-use group presented no significant change [1%, 95% CI = (−2%, 4%), *P* = 0.54].

**Conclusions:** Bisphosphonate use did not increase sclerosis of subchondral bone in established KOA. Bisphosphonate might have a stage-dependent effect on subchondral bone in KOA initiation and progression.

## Introduction

Knee osteoarthritis (KOA) is a joint disease with chronic pain and walking disability that affects 37% of persons older than 60 years old (1, 2). KOA is thus regarded as a serious social issue with substantial economic burdens. KOA involves complex etiologies, such as degeneration of cartilage, abnormal metabolism of subchondral bone, and inflammation of the synovial membrane, caused by genetic, environmental, and biomechanical risk factors.

Subchondral bone is the bony component located distal to calcified cartilage (3, 4), and it has been demonstrated in recent studies to be related to the development and progression of KOA (4, 5). In early KOA, abnormal loading leads to microfractures within the osteochondral bone, and the subsequent healing response involves osteoblasts, osteoclasts, macrophages, mesenchymal stem cells, and complex chemical signals. At this stage, the subchondral remodeling rate increases, and the thickness of the subchondral plate decreases (4, 6). However, in the late stage of KOA, subchondral bone features decreased bone resorption, finally causing bone sclerosis (7). Subchondral sclerosis is one hallmark of KOA and is commonly considered to be accompanied by increased bone volume fraction (BV/TV), trabecular thickness (Tb.Th), trabecular number (Tb.N), and decreased trabecular separation (Tb.Sp) (8). In brief, subchondral bone undergoes distinct pathophysiological processes in the initiation and progression of KOA.

Bisphosphonate has enormous potential for the treatment of KOA. Bisphosphonate can attach to the hydroxyapatite binding sites of bone surfaces. When bisphosphonate contacts the osteoclast, it impairs the ability of the osteoclasts to form the ruffled border that facilitates osteoclasts in adhering to the bony surface and producing the protons that are necessary for bone resorption (9, 10). Therefore, bisphosphonate decreases subchondral bone remodeling, possibly alleviating the increased bone turnover in early KOA. Animal studies have found that bisphosphonate increased BV/TV, Tb.N, and Tb.Th and was beneficial to early KOA (11–13). However, considering that the subchondral bone remodeling process in late KOA was inversely altered, bisphosphonate might negatively affect late KOA. In particular, increased BV/TV, Tb.N, and Tb.Th have been demonstrated to cause established KOA to deteriorate in humans (14–16). The effect of bisphosphonate on the BV/TV, Tb.N, and Tb.Th of subchondral bone in moderate to late KOA was decisive in the justification and safety of bisphosphonate use in these patients, but related studies have been lacking.

The present study aimed to check the safety of bisphosphonate use for subchondral bone in established patients with KOA, that is, whether bisphosphonate use aggravates subchondral bone sclerosis, just as in the theoretical deduction.

## Methods

### Subjects and Study Design

Data in the present study were obtained from the Bone Ancillary Study (BAS), a subproject including 629 subjects of the Osteoarthritis Initiative (OAI) project (https://nda.nih.gov/oai). All BAS participants were members of the progression cohort. The inclusion criteria were as follows: 1. complete baseline and follow-up trabecular MRIs; 2. subchondral bone morphometry data were revealed by the OAI project; 3. radiological presentation of KOA; 4. knee symptoms; and 5. bisphosphonates were used over the previous 30 days or the previous 5 years. Persons with inflammatory arthritis, severe joint space narrowing (JSN) in both knees, unilateral knee joint replacement, severe JSN in the contralateral knee, and the requirement of walking aids for most of the time were excluded. The OAI study was approved by the institutional review boards at corresponding sites, and all participants provided informed consent.

Initial 3-T trabecular MRI and dual-energy X-ray absorptiometry (DXA) were performed at either the 30- or 36-month OAI visit, and then the participants underwent repeat MRI and DXA at the 48-month OAI visit (17). Subchondral bone morphometry was only performed on one knee of each participant (the majority was the right knee). The present study included subjects with both DXA and MRI, and the initial measurement was deemed the baseline.

### Images

Participants were evaluated with a coronal-oblique three-dimensional fast imaging with steady-state free precession (FISP) MRI sequence, which was optimized for the visualization of subchondral trabecular bone (18). The detailed settings are presented in Table 1. FISP acquisition emphasizes the magnetic susceptibility difference and chemical shift differences between marrow fat and the trabeculae. Siemens Trio 3-T MR platforms with a quadrature transmit-receive knee coil (USA Instruments) were used to perform the scans.

**Table 1:** MRI protocol details of 3D FISP (from OAI instruction documents\operations manuals\MRI manuals).

**Parameters**	**Values**
Weighting	Trabecular
Plane	Coronal
Fat Sat	No
Matrix (phase)	512
Matrix (freq)	512
No. of slices	72
FOV (mm)	120
Slice thickness (mm)	1
Skip (mm)	0
Flip Angle (deg)	50
TE/TI (ms)	4.92
TR (ms)	20
BW (Hz/pixel)	180
Chemical shift (pixels)	2.4
NAV (NEX)	1
Echo train length	1
Phase encode axis	R/L
Phase partial fourier (8/8 = 1)	1
Readout partial fourier (8/8 = 1)	1
Slice partial fourier (8/8 = 1)	1
Options:	Elliptical k- space filter and large FOV filter; interpolation to 1024*1024
Distance factor (%)	0
Phase oversampling	0
Slice oversampling	0
Phase resolution	100
Averaging technique	Short term
Gradient rise time	Fast
RF amplitude	Normal
X-resolution (mm)	0.195
Y-resolution (mm)	0.195
Scan time (min)	9.6

### Subchondral Bone Morphometry

Subchondral bone morphometry (BV/TV, Tb.N, Tb.Th, and Tb.Sp) was measured by the established software calcDCN (University of California, San Francisco) (14). A rectangular region of interest (ROI) with a height of 3.75 mm and a width of 14–17 mm depending on the size of the knee was placed in the proximal medial tibia adjacent to the articular cartilage. Only the medial compartment was measured since medial tibiofemoral OA is much more common than lateral OA. Subchondral bone morphometry was calculated in the 20 consecutive MRI slices central to the joint (14).

Bone volume fraction is the percentage of the number of pixels contributing to the bone signal void normalized to the total number of pixels in the ROI. The Tb.Th is determined using the mean value of the mean intercept length for all angles through a given image, measured in millimeters. The Tb.N is calculated by dividing the BV/TV by Tb.Th. Tb.Sp is calculated using the equation (1/Tb.N) - Tb.Th (14, 19). The mean of the metrics across the 20 images within one knee was calculated.

### Bisphosphonate Use

The half-life period of bisphosphonate was reported to be more than 10 years (20). Therefore, this study set two research goals: 1. to determine the effect in those who have ever taken bisphosphonate; and 2. to determine it in those currently taking bisphosphonate.

The participants were asked whether bisphosphonate was taken in the previous 5 years or in the previous 30 days. If the participant used bisphosphonate, the participant was asked about the period during which they took bisphosphonate, when they took a bisphosphonate for the last time, and the name of the bisphosphonate that they took.

### Propensity Score Matching

For the screening of the control group, propensity score matching (PSM) was performed (21). The factors considered to be crucial confounders affecting bone turnover in osteoarthritis were included in the propensity-score algorithm. These factors were determined by investigators and previous reports (22–24). A logistic model with bisphosphonate use as the outcome was used to calculate the propensity score, and the factors included age, body mass index (BMI), sex, baseline bone mineral density (BMD), 12-Item Short Form Survey (SF-12) physical health, Physical Activity Scale for the Elderly (PASE) score, and vitamin D and calcium use in the past 30 days. Then, one participant without bisphosphonate use was matched to each bisphosphonate use subject based on the propensity score. The maximum difference between propensity probabilities for matching was initially set at 0.2 and was adjusted downward if a significant difference in characteristics persisted after matching.

### Sample Size Estimation

The sample size was estimated according to the Hulley et al. equation (25). The yearly change percentage of Tb.Th was used to estimate the sample size. The minimum clinically important difference was set as 5%, and the SD calculated in our pilot study was 0.07. The type I error rate was set as 0.05, and the type II error rate was set as 0.2. The sample size was estimated to be 31 in each group.

### Statistical Analysis

Student's *t*-test was applied to investigate the difference in continuous variables between the groups. The chi-square test was used to test the differences in categorical variables. A two-tailed *p*-value of <0.05 was considered significant. Statistical analysis was conducted with statistical product and service solutions (SPSS) software (version 25.0; SPSS Science, Chicago, IL, USA).

## Results

### Patients Using Bisphosphonate in the Previous 5 Years

Fifty-two patients used bisphosphonate over the previous 5 years, and the average total length of bisphosphonate use was 3.9 years. Before matching, the subjects with bisphosphonate use were significantly older, with less physical activity, and lower BMD at the femoral neck, and there were greater proportions of women, vitamin D use, and calcium use. After PSM was conducted, the baseline characteristics between the bisphosphonate-use group (45 subjects) and the non-bisphosphonate-use group (45 subjects) showed no significant difference (Table 2). Of the 45 participants who had ever used bisphosphonates, 30 patients used only alendronate, seven patients used only risedronate, three patients used alendronate and risedronate, and the other five patients used other bisphosphonates.

**Table 2:** Baseline characteristics of each group before and after propensity score matching (PSM).

	**Subjects using bisphosphonate in previous 5 years**						**Subjects using bisphosphonate in previous 30 days**					
	**Pre-PSM**			**After-PSM**			**Pre-PSM**			**After-PSM**		
	**Non-use**	**Use**	***P***	**Non-use**	**Use**	***P***	**Non-use**	**Use**	***P***	**Non-use**	**Use**	***P***
Number	451	52	-	45	45	-	466	37	-	33	33	-
Age	63.68 ± 9.09	69.5 ± 7.64	<0.05	68.27 ± 8.51	69.24 ± 7.99	0.57	63.94 ± 9.14	68.57 ± 7.78	<0.05	66.91 ± 9.36	68.61 ± 8.13	0.43
BMI	29.53 ± 5.55	27.58 ± 4.41	0.015	28.06 ± 5.16	27.83 ± 4.22	0.81	29.46 ± 5.50	27.68 ± 4.86	<0.05	28.59 ± 5.13	27.65 ± 4.71	0.44
SF-12	45.64 ± 9.76	45.41 ± 11.1	<0.05	46.05 ± 7.44	45.33 ± 11.5	0.72	45.71 ± 9.71	44.38 ± 12.0	0.52	42.58 ± 10.7	44.60 ± 12.4	0.48
PASE	158.09 ± 84.8	109.04 ± 62.0	<0.05	121.2 ± 72.1	111.36 ± 61.4	0.48	156.88 ± 84.5	104.35 ± 60.2	<0.05	104.42 ± 74.6	104.15 ± 57.0	0.98
Hip BMD	0.96 ± 0.17	0.84 ± 0.10	<0.05	0.85 ± 0.14	0.85 ± 0.10	0.85	0.96 ± 0.17	0.83 ± 0.09	<0.05	0.88 ± 0.13	0.83 ± 0.09	0.11
SEX, male%	58%	13%	<0.05	28%	15%	0.12	56%	10%	<0.05	27%	12%	0.12
Vit-D use	31%	71%	<0.05	67%	69%	0.24	33%	70%	<0.05	70%	70%	0.57
Calcium use	42%	83%	<0.05	60%	80%	0.282	43%	81%	<0.05	61%	79%	0.60

*SF-12, 12-Item Short Form Survey physical health; PASE, Physical Activity Scale for the Elderly score; vit-D, vitamin D*.

### Comparison Between the Follow-Up and the Baseline

The Tb.Th significantly increased over the follow-up period in the no-use group [yearly change percentage was 2%, 95% CI = (0%, 3%), *P* <0.05]. No significant change was detected in Tb.Th, BV/TV, Tb.N, or Tb.Sp in the bisphosphonate-use group (Table 3).

**Table 3:** Yearly change of subchondral bone morphometry.

	**Subjects using bisphosphonate in previous 5 years**						**Subjects using bisphosphonate in previous 30 days**					
	**Non-bisphosphonate**			**Use bisphosphonate**			**Non-bisphosphonate**			**Use bisphosphonate**		
	**Mean**	**95%CI**	***P***	**Mean**	**95%CI**	***P***	**Mean**	**95%CI**	***P***	**Mean**	**95%CI**	***P***
BV/TV, percentage	0.05	(−0.04, 0.14)	0.29	0.03	(−0.07, 0.12)	0.59	0.05	(−0.06, 0.16)	0.35	0.05	(−0.06, 0.17)	0.38
Tb.N, percentage	0.02	(−0.06, 0.10)	0.62	0.00	(−0.07, 0.07)	0.94	0.01	(−0.08, 0.10)	0.81	0.02	(−0.06, 0.12)	0.54
Tb.Sp, percentage	0.09	(−0.01, 0.19)	0.08	0.10	(−0.00, 0.19)	0.05	0.11	(−0.02, 0.25)	0.10	0.05	(−0.05, 0.16)	0.29
Tb.Th, percentage	**0.02**	**(0.00, 0.03)**	** <0.05**	0.01	(−0.01, 0.03)	0.44	**0.02**	**(0.01, 0.04)**	**0.01**	0.01	(−0.02, 0.04)	0.54

*Bold value means statistical significance*.

### Comparisons Between Non-bisphosphonate and Bisphosphonate Use

**Figure 1:**
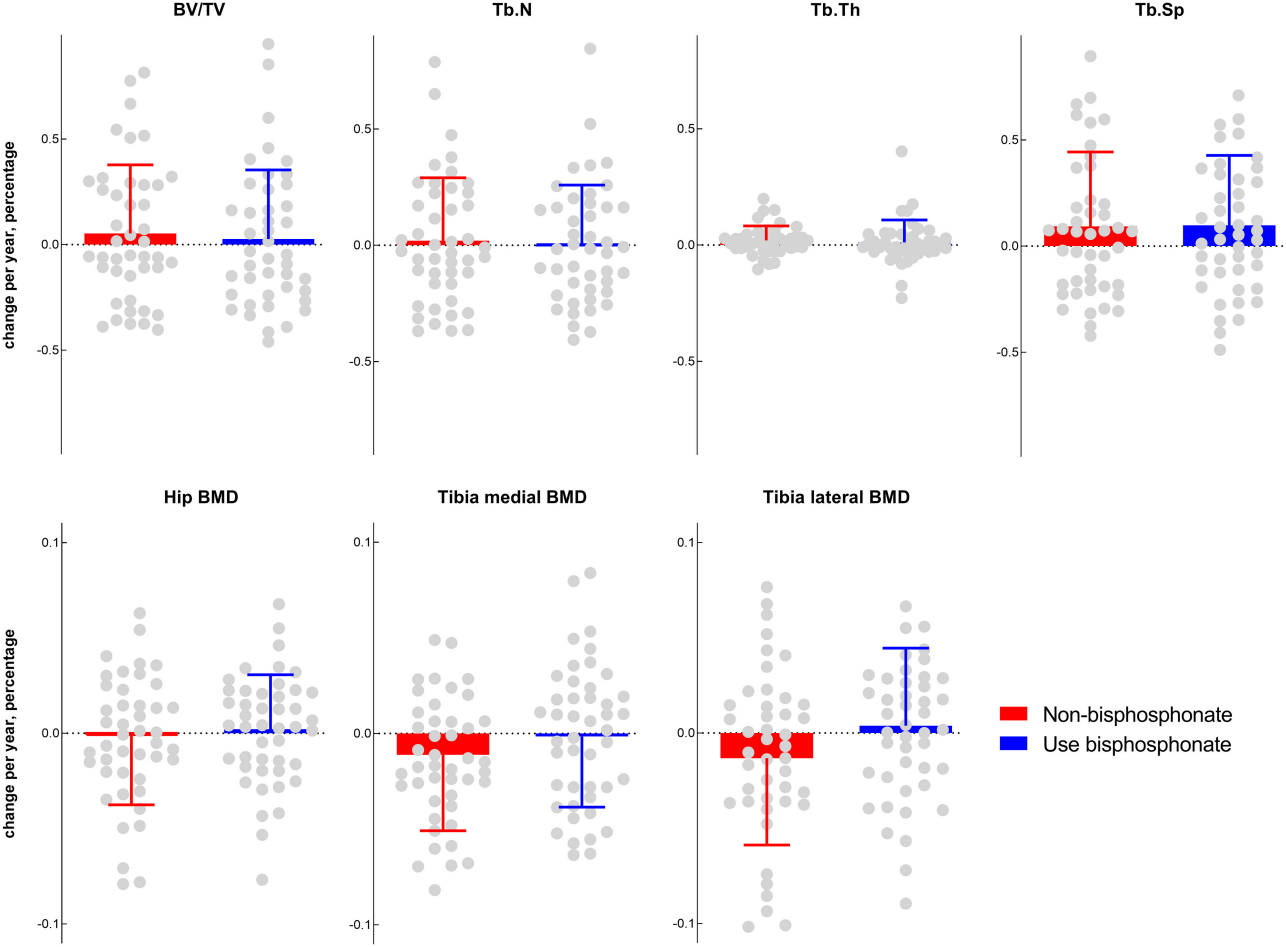
Comparisons of subchondral bone morphometry and BMD between the bisphosphonate ever-use (in the previous 5 years) group and the non-bisphosphonate group.

The mean annual percentage changes between the non-bisphosphonate-use group and the bisphosphonate-use group were compared to evaluate the effect of bisphosphonate. All outcomes (BV/TV, Tb.N, Tb.Sp, Tb.Th, tibia medial BMD, tibia lateral BMD, and hip BMD) between the two groups presented no significant differences (Figure 1).

### Pain and Function Scores

The pain score (numerical rating scale) at baseline and the pain change over 1 year between the two groups were not significantly different. The difference in the western ontario and McMaster universities osteoarthritis index (WOMAC) score was also not significant (Table 4).

**Table 4:** Pain score and WOMAC total score in the participants.

	**Subjects using bisphosphonate in previous 5 years**			**Subjects using bisphosphonate in previous 30 days**		
	**Non-use**	**Use**	***P***	**Non-use**	**Use**	***P***
Baseline pain score	2.73 ± 2.85	3.35 ± 2.37	0.26	3.05 ± 2.87	3.29 ± 2.27	0.70
The change of pain score	0.77 ± 3.19	0.35 ± 2.72	0.50	0 ± 3.04	0.08 ± 2.99	0.90
Baseline WOMAC total score	15.39 ± 15.96	15.94 ± 13.57	0.86	14.97 ± 15.96	15.22 ± 13.60	0.94
The change of total score	−0.52 ± 11.78	0.12 ± 10.69	0.78	−4.01 ± 12.01	0.37 ± 11.23	0.12

### Patients Using Bisphosphonate in the Previous 30 Days

**Figure 2:**
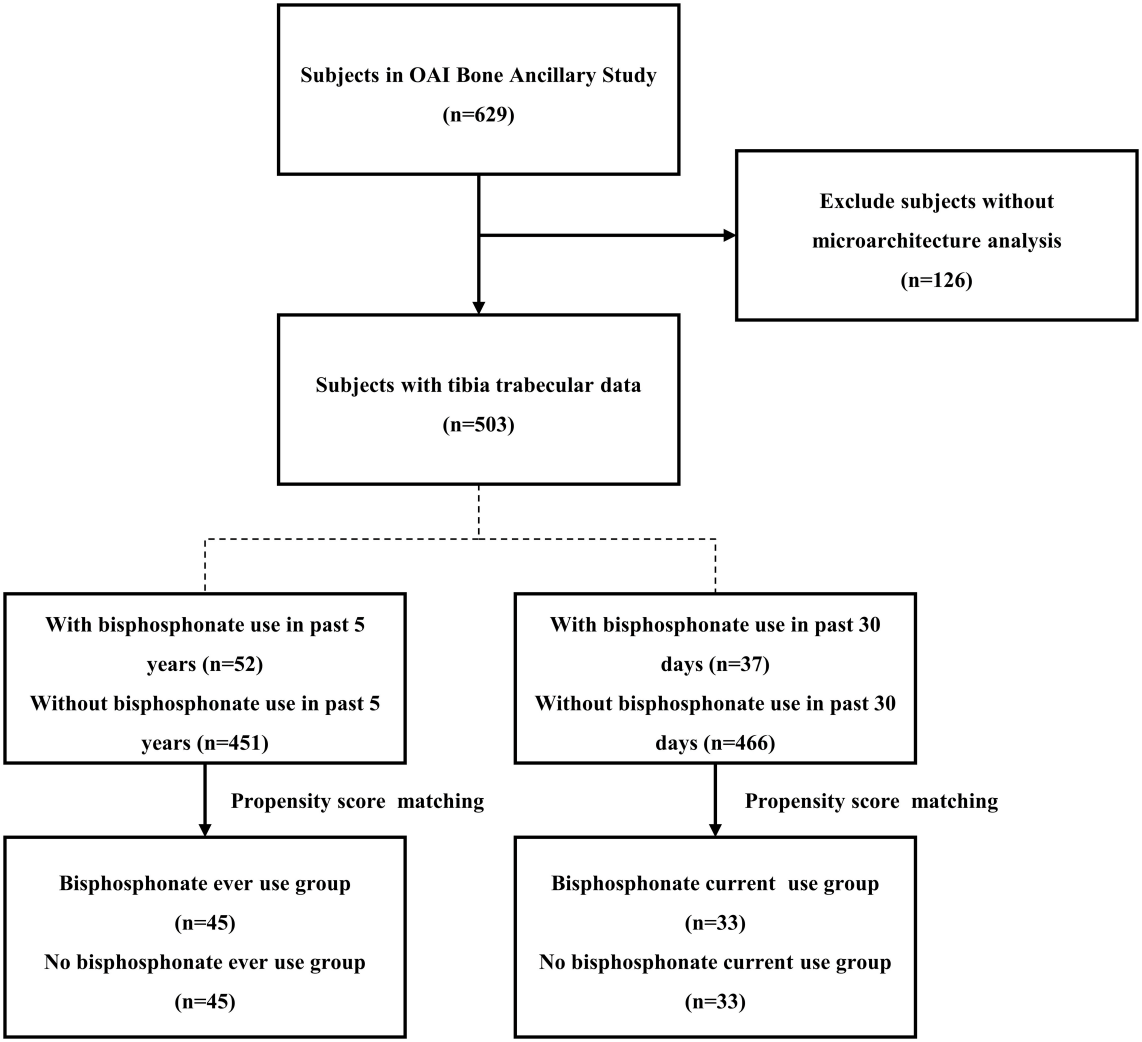
The flowchart for screening participants.

Thirty-seven patients used bisphosphonate in the previous 30 days, and they were deemed current users (Figure 2). The 37 current users were included among the 52 ever users, and we established ever-use and current-use groups because it is unknown how long the effect of bisphosphonate on subchondral bone lasts. After matching, 34 participants were currently using bisphosphonates, 24 patients used only alendronate, six patients used only risedronate, two patients used alendronate and risedronate, and one patient used other bisphosphonates.

### Comparison Between the Follow-Up and the Baseline

The Tb.Th significantly increased over the follow-up period in the no-use group [yearly change percentage was 2%, 95% CI = (1%, 4%), *P* <0.05]. No significant change was detected in Tb.Th, BV/TV, Tb.N, or Tb.Sp in the bisphosphonate-use group (Table 3).

### Comparisons Between Non-bisphosphonate and Bisphosphonate Use

**Figure 3:**
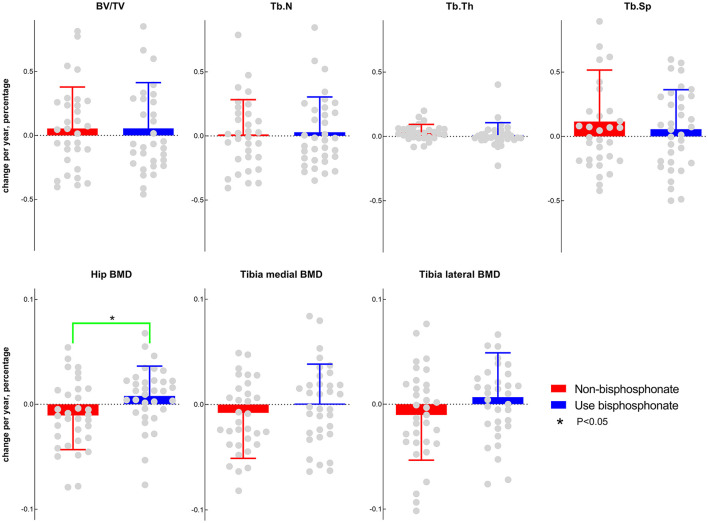
Comparisons of subchondral bone morphometry and BMD between the bisphosphonate current-use (in the previous 30 days) group and the non-bisphosphonate group.

The yearly percentage change in hip BMD in the bisphosphonate-use group was significantly higher than that in the non-bisphosphonate-use group (0.7 vs. −1%, *P* = 0.02), while the other outcomes (BV/TV, Tb.N, Tb.Sp, Tb.Th, tibia medial BMD, and tibia lateral BMD) between the two groups presented no significant differences (Figure 3).

### Pain and Function Scores

Both the pain score (numerical rating scale) and the WOMAC score showed no significant difference between the groups (Table 4).

## Discussions

The effect of bisphosphonate on the subchondral bone morphometry of patients with KOA remains unclear. The concern about the possibility that bisphosphonate might increase the sclerosis of subchondral bone might discourage clinicians from using bisphosphonate for established KOA. The present study found that bisphosphonate use did not alter tibial subchondral bone morphometry, which indicates sclerosis.

Fernández-Martín's research (26) also revealed that bisphosphonate did not alter subchondral bone morphometry. However, there were enormous differences between the research settings. First, our results were from human data, which were different from previous studies using animal models. The mechanism of KOA in animal models could differ from that in humans; in particular, most animal models adopt traumatic KOA, which is not in accord with most cases of practical human KOA. Second, our study only enrolled established patients with KOA to investigate the effect of bisphosphonate on the late stage of KOA, while Fernández-Martín's research studied rabbits in the early stage.

The difference in the subchondral bone morphometry change was not significant between the two groups, but the hip BMD change in the current bisphosphonate-use group was significantly greater than that in the no current bisphosphonate-use group. There seems to be a contradiction between increased BMD and unchanged subchondral bone morphometry or sclerosis. This contradiction might be explained by the difference between increased trabecular volume and increased BMD. Li et al. (27) demonstrated that the subchondral bone of patients with OA contained greater bone volume but less mineralization than disease-free subchondral bone. In late KOA, bone volume (or sclerosis) increases due to decreased bone resorption and relatively increased bone formation. However, due to the lack of sufficient time for full mineralization in fast bone formation, the material density of subchondral bone will decrease (4).

The effect of bisphosphonate on patients with KOA has been widely investigated, but the results have been conflicting. In Maurizio Rossini et al.'s double-blind, phase III, randomized clinical trial (RCT) (28), intra-articular injection of 2 mg of clodronate was associated with a lower visual analogue scale (VAS) and less analgesic drug use in patients with KOA at the 5-week follow-up. Laslett et al. (29) reported a significant reduction in numeric rating scale pain in the first 3 years with bisphosphonate use in patients with KOA. Arti et al. (30) conducted a RCT of 130 patients, demonstrating that a combination therapy of glucosamine and alendronate significantly improved stiffness and function compared with glucosamine alone. Laslett et al. (31) revealed that zoledronic acid reduced knee pain and bone marrow lesion size over 6 months. One hypothesis was that the analgesic effect of bisphosphonate arose from the suppression of proton secretion by osteoclasts. In situations of high bone turnover with increased osteoclast activity, the acidification of the osteoclast environment was increased by the greater level of secreted protons, which would lead to the activation of acid-sensing receptors, such as transient receptor potential vanilloid subtype 1 (TRPV1) channels and acid-sensing ion channel 2 or 3 (ASIC2/3), which are located on bone-innervating primary afferents, producing nociceptive signaling (32). The effect of bisphosphonate on KOA structural progression might occur through bone remodeling (33). However, some studies have argued against the use of bisphosphonate in treating KOA. Cai et al. (34) detected no significant difference between the zoledronic acid and placebo groups regarding changes in cartilage volume, visual analog scale, or bone marrow lesion size. Ballal et al. (35) also reported that no benefit to bone marrow lesions (BML) volume was observed over a 12-month period. It is known that enlarging BMLs can lead to greater cartilage loss (36). Our study provided additional evidence on this issue from the perspective of morphometry.

Subchondral bone morphometry was demonstrated to be related to the severity and progression of KOA in humans. Chiba et al. (16) examined 60 patients with KOA with 3-T MRI and found that medial tibial subchondral BV/TV, Tb.N, and Tb.Th increased as the cartilage area decreased in the medial compartment. Lo et al. (14) demonstrated that BV/TV, Tb.N, and Tb.Th were positively related to medial JSN scores, and Tb.Sp was negatively related to medial JSN scores. Lo et al. (15) also reported that higher baseline BV/TV, Tb.N, and Tb.Th and lower baseline Tb.Sp were associated with more progression of JSN in the medial compartment at 12 months. Therefore, BV/TV, Tb.N, Tb.Th, and Tb.Sp are excellent indicators for both subchondral bone remodeling and KOA progression.

Our results of no significant improvement of morphometry in the bisphosphonate group could not conclude that bisphosphonate was useless for KOA because the molecular subtypes of KOA were not considered in the participants in this research. Vaysbrot's meta-analysis (37) proposed that bisphosphonate might be beneficial in certain subsets of patients who display high rates of subchondral bone turnover. Lv et al. (38) proposed that progressive KOA could be divided into four subtypes based on pathophysiology: cartilage degradation-driven, bone remodeling-driven, inflammation-driven, and pain-driven subtypes. Theoretically, bisphosphonate is most beneficial to the bone remodeling-driven KOA subtype. Therefore, future studies enrolling the corresponding subtype *via* strict inclusion criteria considering molecular biomarkers remain necessary. However, it might also be oversimplified to divide KOA into a high turnover subtype and other subtypes. Bone turnover in one person might change with the pathological state. Furthermore, it remains unknown whether subchondral bone changes are a trigger factor or a consequence of KOA. Lv et al.'s classification considered increased resorption to be a trigger factor and supported bisphosphonate therapy. However, when the bone turnover of patients with KOA changes from high to low, the role of bisphosphonates might also change from beneficial to detrimental. Decreased resorption could also be seen as a consequence of late KOA, and from this perspective, bisphosphonate might be harmful and cause sclerosis. Our research partially resolved this concern since we found that, in late-stage KOA, the use of bisphosphonates did not increase the sclerosis of subchondral bone. In brief, our results did not support the use of bisphosphonate in KOA therapy itself, but we considered that the use of bisphosphonate for patients with osteoporosis who also had KOA would not exacerbate KOA.

The significance of the present study was in two aspects. The concern about potential sclerosis of subchondral bone after bisphosphonate use was addressed to some extent. Since there is no applicable method in clinical practice to predict when and how bone remodeling is altered from increased resorption in the early stage to decreased resorption in the late stage, our results demonstrated that bisphosphonate did not increase the risk of subchondral bone sclerosis in moderate to late KOA, which might prolong the therapy window of bisphosphonate for its analgesic effect on KOA. The present study also revealed the difference in the effect of bisphosphonate on the subchondral bone between late KOA and animal KOA models, perhaps providing some insight into the different pharmacological mechanisms of bisphosphonate in KOA initiation and progression. The microenvironment alteration in late KOA, such as the cross talk between synovium inflammation and subchondral bone after fissure form in cartilage, might play a crucial role in the effect of bisphosphonate on subchondral bone.

There were several limitations to the present study. First, intraarticular injection of bisphosphonate might have greater potential than oral bisphosphonate for KOA therapy. The results of this study could not substitute for the change after intra-articular injection of bisphosphonate. Second, due to the limited sample size, no more detailed classification of bisphosphonates or analysis was conducted. Third, the pattern of decreased resorption in late KOA might not be identical in each patient, and different subtypes of KOA might exist. We failed to classify the participants by osteoclast function or molecular markers due to the absence of related data in the OAI project. Fourth, the exact regimen of bisphosphonate for each participant was unknown, and further RCTs are necessary.

## Conclusions

The patients with KOA who used bisphosphonate did not experience greater sclerosis of tibial subchondral bone than patients who did not use bisphosphonate. Considering that previous studies found that bisphosphonate in early KOA significantly altered subchondral bone morphometry, this study suggests that the effect of bisphosphonate on tibial subchondral bone in the initiation and progression of KOA might be OA-stage dependent.

## Data Availability Statement

The original contributions presented in the study are included in the article/supplementary material, further inquiries can be directed to the corresponding author/s.

## Ethics Statement

Ethical review and approval was not required for the study on human participants in accordance with the local legislation and institutional requirements. The patients/participants provided their written informed consent to participate in this study. All OAI project participants signed the informed consent.

## Author Contributions

ML, YN, and BS: study design and manuscript writing. YW, YZ, and JX: data extracting. LW and YL: statistical analysis. ML and BS: data checking. All authors contributed to the article and approved the submitted version.

## Funding

This study was funded by National Natural Science Foundation of China (Program No. 81974347), National Clinical Research Center for Geriatrics, West China Hospital, Sichuan University (No. Z20192003), and Science and Technology of Foundation of Sichuan province of China (2021YFH0094). Post-Doctor Research Project, West China Hospital, Sichuan University (No. 2020HXBH081). All authors declared that the funding did not have any effect on the results of this study.

## Conflict of Interest

The authors declare that the research was conducted in the absence of any commercial or financial relationships that could be construed as a potential conflict of interest.

## Publisher's Note

All claims expressed in this article are solely those of the authors and do not necessarily represent those of their affiliated organizations, or those of the publisher, the editors and the reviewers. Any product that may be evaluated in this article, or claim that may be made by its manufacturer, is not guaranteed or endorsed by the publisher.

## References

[1] Dillon CF Hirsch R Rasch EK Gu Q. Symptomatic hand osteoarthritis in the United States: prevalence and functional impairment estimates from the third US National Health and Nutrition Examination Survey, 1991–1994. Am J Phys Med Rehabil. (2007) 86:12–21. 10.1097/PHM.0b013e31802ba28e 17304684

[2] Sharma L. Osteoarthritis of the knee. N Engl J Med. (2021) 384:51–9. 10.1056/NEJMcp1903768 33406330

[3] Madry H van Dijk CN Mueller-Gerbl M. The basic science of the subchondral bone. Knee Surg Sports Traumatol Arthrosc. (2010) 18:419–33. 10.1007/s00167-010-1054-z 20119671

[4] Burr DB Gallant MA. Bone remodelling in osteoarthritis. Nat Rev Rheumatol. (2012) 8:665–73. 10.1038/nrrheum.2012.130 22868925

[5] Weber A Chan PMB Wen C. Do immune cells lead the way in subchondral bone disturbance in osteoarthritis? Prog Biophys Mol Biol. (2019) 148:21–31. 10.1016/j.pbiomolbio.2017.12.004 29277342

[6] Intema F Sniekers YH Weinans H Vianen ME Yocum SA Zuurmond AM. Similarities and discrepancies in subchondral bone structure in two differently induced canine models of osteoarthritis. J Bone Miner Res. (2010) 25:1650–7. 10.1002/jbmr.39 20200954

[7] Karsdal MA Leeming DJ Dam EB Henriksen K Alexandersen P Pastoureau P. Should subchondral bone turnover be targeted when treating osteoarthritis? Osteoarthritis Cartilage. (2008) 16:638–46. 10.1016/j.joca.2008.01.014 18362080

[8] Kamibayashi L Wyss UP Cooke TD Zee B. Trabecular microstructure in the medial condyle of the proximal tibia of patients with knee osteoarthritis. Bone. (1995) 17:27–35. 10.1016/8756-3282(95)00137-3 7577155

[9] Sato M Grasser W Endo N Akins R Simmons H Thompson D. Bisphosphonate action. Alendronate localization in rat bone and effects on osteoclast ultrastructure. J Clin Investig. (1991) 88:2095–105. 10.1172/JCI115539 1661297 PMC295810

[10] Colucci S Minielli V Zambonin G Cirulli N Mori G Serra M. Alendronate reduces adhesion of human osteoclast-like cells to bone and bone protein-coated surfaces. Calcif Tissue Int. (1998) 63:230–5. 10.1007/s002239900519 9701627

[11] Mohan G Perilli E Parkinson IH Humphries JM Fazzalari NL Kuliwaba JS. Pre-emptive, early, and delayed alendronate treatment in a rat model of knee osteoarthritis: effect on subchondral trabecular bone microarchitecture and cartilage degradation of the tibia, bone/cartilage turnover, and joint discomfort. Osteoarthritis Cartilage. (2013) 21:1595–604. 10.1016/j.joca.2013.06.020 23827368

[12] Hainan C Qirong D Wei J. 3-D structural changes in subchondral bone and the effect of bisphosphonate intervention in early osteoarthritis. Cambios Estructurales Tridimensionales del Hueso Subcondral y el Efecto de la Intevención con Bisfosfonato en la Osteoartritis Temprana. (2016) 34:291–7. 10.4067/S0717-95022016000100042

[13] Hainan Chen WJ. Qirong D, Kan Y. Finite element analysis of biomechanical variation of subchondral bone in osteoarthritis. Kuwait Med J. (2020) 52:7. Available online at: https://www.webofscience.com/wos/alldb/full-record/WOS:000540762300010.

[14] Lo GH Tassinari AM Driban JB Price LL Schneider E Majumdar S. Cross-sectional DXA and MR measures of tibial periarticular bone associate with radiographic knee osteoarthritis severity. Osteoarthritis Cartilage. (2012) 20:686–93. 10.1016/j.joca.2012.03.006 22430052 PMC3760173

[15] Lo GH Schneider E Driban JB Price LL Hunter DJ Eaton CB. Periarticular bone predicts knee osteoarthritis progression: data from the Osteoarthritis Initiative. Semin Arthritis Rheum. (2018) 48:155–61. 10.1016/j.semarthrit.2018.01.008 29449014 PMC6853601

[16] Chiba K Uetani M Kido Y Ito M Okazaki N Taguchi K. Osteoporotic changes of subchondral trabecular bone in osteoarthritis of the knee: a 3-T MRI study. Osteoporos Int. (2012) 23:589–97. 10.1007/s00198-011-1585-2 21359670

[17] MacKay JW Kapoor G Driban JB Lo GH McAlindon TE Toms AP. Association of subchondral bone texture on magnetic resonance imaging with radiographic knee osteoarthritis progression: data from the Osteoarthritis Initiative Bone Ancillary Study. Eur Radiol. (2018) 28:4687–95. 10.1007/s00330-018-5444-9 29721684 PMC6182744

[18] Schneider E Lo GH Sloane G Fanella L Hunter DJ Eaton CB. Magnetic resonance imaging evaluation of weight-bearing subchondral trabecular bone in the knee. Skeletal Radiol. (2011) 40:95–103. 10.1007/s00256-010-0943-z 20449585 PMC3886640

[19] Majumdar S Newitt D Jergas M Gies A Chiu E Osman D. Evaluation of technical factors affecting the quantification of trabecular bone structure using magnetic resonance imaging. Bone. (1995) 17:417–30. 10.1016/S8756-3282(95)00263-4 8573417

[20] Gertz BJ Holland SD Kline WF Matuszewski BK Porras AG. Clinical pharmacology of alendronate sodium. Osteoporos Int. (1993) 3(Suppl.3):S13–16. 10.1007/BF01623002 8298197

[21] D'Agostino Jr RB. Propensity score methods for bias reduction in the comparison of a treatment to a non-randomized control group. Statist Med. (1998) 17:2265–81. 10.1002/(SICI)1097-0258(19981015)17:19<2265::AID-SIM918>3.0.CO 9802183

[22] Bainbridge KE Sowers M Lin X Harlow SD. Risk factors for low bone mineral density and the 6-year rate of bone loss among premenopausal and perimenopausal women. Osteoporosis Int. (2004) 15:439–46. 10.1007/s00198-003-1562-5 15205714

[23] Daly RM Gianoudis J Kersh ME Bailey CA Ebeling PR Krug R. Effects of a 12-month supervised, community-based, multimodal exercise program followed by a 6-month research-to-practice transition on bone mineral density, trabecular microarchitecture, and physical function in older adults: a randomized controlled trial. J Bone Mineral Res. (2020) 35:419–29. 10.1002/jbmr.3865 31498937

[24] Di Gregorio S Del Rio L Rodriguez-Tolra J Bonel E García M Winzenrieth R. Comparison between different bone treatments on areal bone mineral density (aBMD) and bone microarchitectural texture as assessed by the trabecular bone score (TBS). Bone. (2015) 75:138–43. 10.1016/j.bone.2014.12.062 25571842

[25] Hulley SBCS Browner WS Grady D Newman TB. Designing Clinical Research: An Epidemiologic Approach. 4th ed. Philadelphia, PA: Lippincott Williams & Wilkins (2013). Appendix 6B:75.

[26] Fernández-Martín S Permuy M López-Peña M Muñoz F González-Cantalapiedra A. No effect of long-term risedronate use on cartilage and subchondral bone in an experimental rabbit model of osteoarthritis. Front Vet Sci. (2020) 7:576212. 10.3389/fvets.2020.576212 33240955 PMC7667022

[27] Li G Yin J Gao J Cheng TS Pavlos NJ Zhang C. Subchondral bone in osteoarthritis: insight into risk factors and microstructural changes. Arthritis Res Ther. (2013) 15:223. 10.1186/ar4405 24321104 PMC4061721

[28] Rossini M Adami S Fracassi E Viapiana O Orsolini G Povino MR. Effects of intra-articular clodronate in the treatment of knee osteoarthritis: results of a double-blind, randomized placebo-controlled trial. Rheumatol Int. (2015) 35:255–63. 10.1007/s00296-014-3100-5 25080876

[29] Laslett LL Kingsbury SR Hensor EM Bowes MA Conaghan PG. Effect of bisphosphonate use in patients with symptomatic and radiographic knee osteoarthritis: data from the Osteoarthritis Initiative. Ann Rheum Dis. (2014) 73:824–30. 10.1136/annrheumdis-2012-202989 23585518

[30] Arti HR Azemi ME. Comparing the effect of glucosamine and glucosamine with alendronate in symptomatic relieve of degenerative knee joint disease: a double- blind randomized clinical trial study. Jundishapur J Nat Pharm Prod. (2012) 7:87–92. 10.5812/jjnpp.3405 24624161 PMC3941844

[31] Laslett LL Doré DA Quinn SJ Boon P Ryan E Winzenberg TM. Zoledronic acid reduces knee pain and bone marrow lesions over 1 year: a randomised controlled trial. Ann Rheum Dis. (2012) 71:1322–8. 10.1136/annrheumdis-2011-200970 22355040

[32] Tzschentke TM. Pharmacology of bisphosphonates in pain. Br J Pharmacol. (2021) 178:1973–94. 10.1111/bph.14799 31347149

[33] Tateiwa D Yoshikawa H Kaito T. Cartilage and bone destruction in arthritis: pathogenesis and treatment strategy: a literature review. Cells. (2019) 8:80818. 10.3390/cells8080818 31382539 PMC6721572

[34] Cai G Aitken D Laslett LL Pelletier JP Martel-Pelletier J Hill C. Effect of intravenous zoledronic acid on tibiofemoral cartilage volume among patients with knee osteoarthritis with bone marrow lesions: a randomized clinical trial. J Am Med Assoc. (2020) 323:1456–66. 10.1001/jama.2020.2938 32315057 PMC7175085

[35] Ballal P Sury M Lu N Duryea J Zhang Y Ratzlaff C. The relation of oral bisphosphonates to bone marrow lesion volume among women with osteoarthritis. Osteoarthritis Cartilage. (2020) 28:1325–9. 10.1016/j.joca.2020.07.006 32768598 PMC7530037

[36] Cai G Aitken D Laslett LL Hill C Wluka AE March L. The association between change in bone marrow lesion size and change in tibiofemoral cartilage volume and knee symptoms. Rheumatology. (2021) 60:2791–800. 10.1093/rheumatology/keaa716 33253381

[37] Vaysbrot EE Osani MC Musetti MC McAlindon TE Bannuru RR. Are bisphosphonates efficacious in knee osteoarthritis? A meta-analysis of randomized controlled trials. Osteoarthritis Cartilage. (2018) 26:154–64. 10.1016/j.joca.2017.11.013 29222056

[38] Lv Z Yang YX Li J Fei Y Guo H Sun Z. Molecular classification of knee osteoarthritis. Front Cell Dev Biol. (2021) 9:725568. 10.3389/fcell.2021.725568 34513847 PMC8429960

